# The Level of Protein in Milk Formula Modifies Ileal Sensitivity to LPS Later in Life in a Piglet Model

**DOI:** 10.1371/journal.pone.0019594

**Published:** 2011-05-09

**Authors:** Livie Chatelais, Agnès Jamin, Christèle Gras-Le Guen, Jean-Paul Lallès, Isabelle Le Huërou-Luron, Gaëlle Boudry

**Affiliations:** 1 INRA, UMR 1079 SENAH, Saint-Gilles, France; 2 Agrocampus Ouest, Rennes, France; 3 Pôle Médico-Chirurgical de Pédiatrie et de Génétique Clinique, CHU Rennes, Rennes, France; 4 Service de Réanimation Pédiatrique, Hôpital Mère - Enfant, CHU Nantes, Nantes, France; Paris Institute of Technology for Life, Food and Environmental Sciences, France

## Abstract

**Background:**

Milk formulas have higher protein contents than human milk. This high protein level could modify the development of intestinal microbiota, epithelial barrier and immune functions and have long-term consequences.

**Methodology/Principal findings:**

We investigated the effect of a high protein formula on ileal microbiota and physiology during the neonatal period and later in life. Piglets were fed from 2 to 28 days of age either a normoprotein (NP, equivalent to sow milk) or a high protein formula (HP, +40% protein). Then, they received the same solid diet until 160 days. During the formula feeding period ileal microbiota implantation was accelerated in HP piglets with greater concentrations of ileal bacteria at d7 in HP than NP piglets. Epithelial barrier function was altered with a higher permeability to small and large probes in Ussing chambers in HP compared to NP piglets without difference in bacterial translocation. Infiltration of T cells was increased in HP piglets at d28. IL-1β and NF-κB sub-units mRNA levels were reduced in HP piglets at d7 and d28 respectively; plasma haptoglobin also tended to be reduced at d7. Later in life, pro-inflammatory cytokines secretion in response to high doses of LPS in explants culture was reduced in HP compared to NP piglets. Levels of mRNA coding the NF-κB pathway sub-units were increased by the challenge with LPS in NP piglets, but not HP ones.

**Conclusions/Significance:**

A high protein level in formula affects the postnatal development of ileal microbiota, epithelial barrier and immune function in piglets and alters ileal response to inflammatory mediators later in life.

## Introduction

Although human breast milk is the optimal nutrition during infancy, many infants are still formula-fed for practical or medical reasons. Milk formula composition has been improved during the last decades to tend to resemble human milk. Cow's milk protein is the major source of protein in formulas but due to difference in protein and amino acid digestibility, bioavailability and efficiency of utilization between human milk and formula, the amount of protein per energy content has generally been higher in formula than in human milk to meet the protein and amino acid requirements of infants (up to 2.5 g/100 kcal for the formula, i.e. up to 40% more proteins than in human breast milk) [1], [2]. Although the current tendency is to reduce protein level in formula for full term healthy babies, numerous babies have been fed such formula for years during the last decades. The use of high protein formula is still encouraged in at risk populations such as low birth weight babies who had suffered intra-uterine growth restriction to ensure a rapid post-natal catch up growth [3], [4]. Yet, such nutritional practice seems to have long-term metabolic consequences [5], [6].

At the intestinal level, the interplay between gut bacterial colonization, epithelial barrier and gut associated-lymphoid tissue (GALT) plays a crucial role in the development of intestinal function and maturation of the immune system during the first months of life in neonates. The type of bacteria colonizing the intestine of newborns and the timing determine the immunomodulation of the naïve GALT [7]. Studies in infants with prebiotics and probiotics suggest that manipulations of the microbiota early in life affect the immune system [8]. The physical barrier formed by the intestinal epithelium is also crucial since bacterial translocation has been shown to be required for the neonate to achieve GALT development [9]. This interplay can be modulated by a fourth factor, namely food. Indeed, food components may be the source of antigens to which the immune system must become tolerant and antigens might themselves modulate immune maturation. Food also provides factors or nutrients that influence the intestinal microbiota and epithelial barrier function, which in turn will affect antigen exposure, immune maturation and immune function [10].

Although high protein formulas have been or are still commonly used in neonates, the role of high levels of protein as modulators of intestinal microbiota and epithelial and immune cells has been poorly investigated. In adults, a high protein diet has been shown to increase lactic acid bacteria in ileal digesta of human adults [11] and supplementation of the diet with a mixture of amino acids increases the total number of faecal bacteria in a model of colitis in adult rats [12]. Specific amino acids such as glutamine have been shown to enhance barrier function in cell culture or animal models [13]. Finally, presence of proteins and therefore antigenic structures in the diet has been shown to be essential in the maturation of the GALT in mice [14]. We hypothesized that, as described in adults, a high level of protein in infant formula will modulate intestinal microbiota and non-immune and immune intestinal barrier development. Moreover, regarding the key role of the neonatal period in GALT maturation, we also hypothesized that early modifications of intestinal ecology and physiology would impact intestinal sensitivity to inflammation later in life. We therefore compared the postnatal development of ileal microbiota, epithelial permeability and immune function of piglets fed two types of formula differing in their protein content: close to sow milk or with 40% more protein per unit of energy. In a follow-up experiment we evaluated the long-term effects of this high protein formula on intestinal sensitivity to inflammation.

## Results

### Piglet growth

The mean protein intake over the whole experimental period (d 2–d 28) in the first experiment was 35% higher for HP compared to NP piglets (17.5±0.2 vs 12.6±0.2 g.d^−1^.kg of metabolic weight^−1^, *P*<0.001) but with similar energy intakes (1165±9 vs 1183±14 kJ.d^−1^.kg of metabolic weight^−1^, *P*>0.05) as expected. The HP diet enhanced piglet growth from the 16^th^ day of life, with a 19% higher daily weight gain relative to the initial birth weight in HP compared to NP piglets (188±10 vs 158±10 g.d^−1^.kg of birth weight^−1^, *P* = 0.02) over the whole experimental period.

Similar increase in daily weight gain during the formula-feeding period was observed with HP piglets in the follow-up experiment (data not shown). After weaning, no difference in feed intake and daily weight gain was observed between the two groups of animals (feed intake: NP 46±1 vs HP: 45±1 g.kg^−1^.d^−1^, P>0.05; daily weight gain: NP 755±11 vs HP 751±11 g.d^−1^, P>0.05). The final body weight at slaughter did not differ between groups (NP 116±8 vs HP 114±7 kg, P>0.05).

### Ileal microbiota during the neonatal period

To test our hypothesis of a stimulatory effect of the HP formula on microbiota growth, we measured the concentration of aerobic and anaerobic bacteria in ileal contents. A higher concentration of total aerobes and anaerobes in the ileal contents of HP compared to NP piglets was observed at d 7 (Table 1). Mucosal-adherent bacteria were then analysed to evaluate if this higher concentration of bacteria induced an increase in the number of bacteria actually in contact with the mucosa. However, no change in the total number of aerobes or anaerobes bacteria adherent to the ileal mucosa was observed (Table 1). Nonetheless, while the concentration of *Lactobacillus* was increased in ileal contents, the number of *Lactobacillus* adherent to the mucosa was lower in HP than NP piglets at d 7 (Table 1).

**Table 1:** Microbiota in the ileal content or adherent to the ileal mucosa of NP and HP piglets at d2, d7 and d28.

	d2	d7		d28	
*log CFU.g^−1^*		NP	HP	NP	HP
Content					
Total aerobes	7.0±0.4	6.5±0.5	8.7±0.5*	8.9±0.2	9.2±0.2
Total anaerobes	6.9±0.4	6.8±0.5	8.9±0.3*	8.9±0.1	9.0±0.1
*E.coli*	5.9±0.3	6.2±0.5	8.2±0.5*	9.0±0.7	8.7±0.2
*Enterococcus*	2.8±0.8	2.6±0.6	4.5±1.5	4.4±1.4	4.5±1.6
*Lactobacillus*	6.9±0.4	6.3±0.5	8.7±0.3*	8.7±0.1	8.8±0.1
Adherent					
Total aerobes	6.8±0.3	7.6±0.6	7.0±0.3	7.1±0.4	6.9±0.7
Total anaerobes	6.4±0.2	7.4±0.5	7.2±0.3	7.4±0.4	5.3±1.9
*E.coli*	5.9±0.4	7.1±0.6	7.0±0.5	6.7±0.4	6.6±0.7
*Enterococcus*	2.0±0.0	5.0±1.5	4.0±0.9	4.7±1.2	4.0±1.4
*Lactobacillus*	6.4±0.2	7.3±0.5	5.8±0.3*	5.6±1.0	5.5±1.3

Values are means ± SE, n = 6.

*P<0.05.

### Epithelial barrier function during the neonatal period

**Figure 1:**
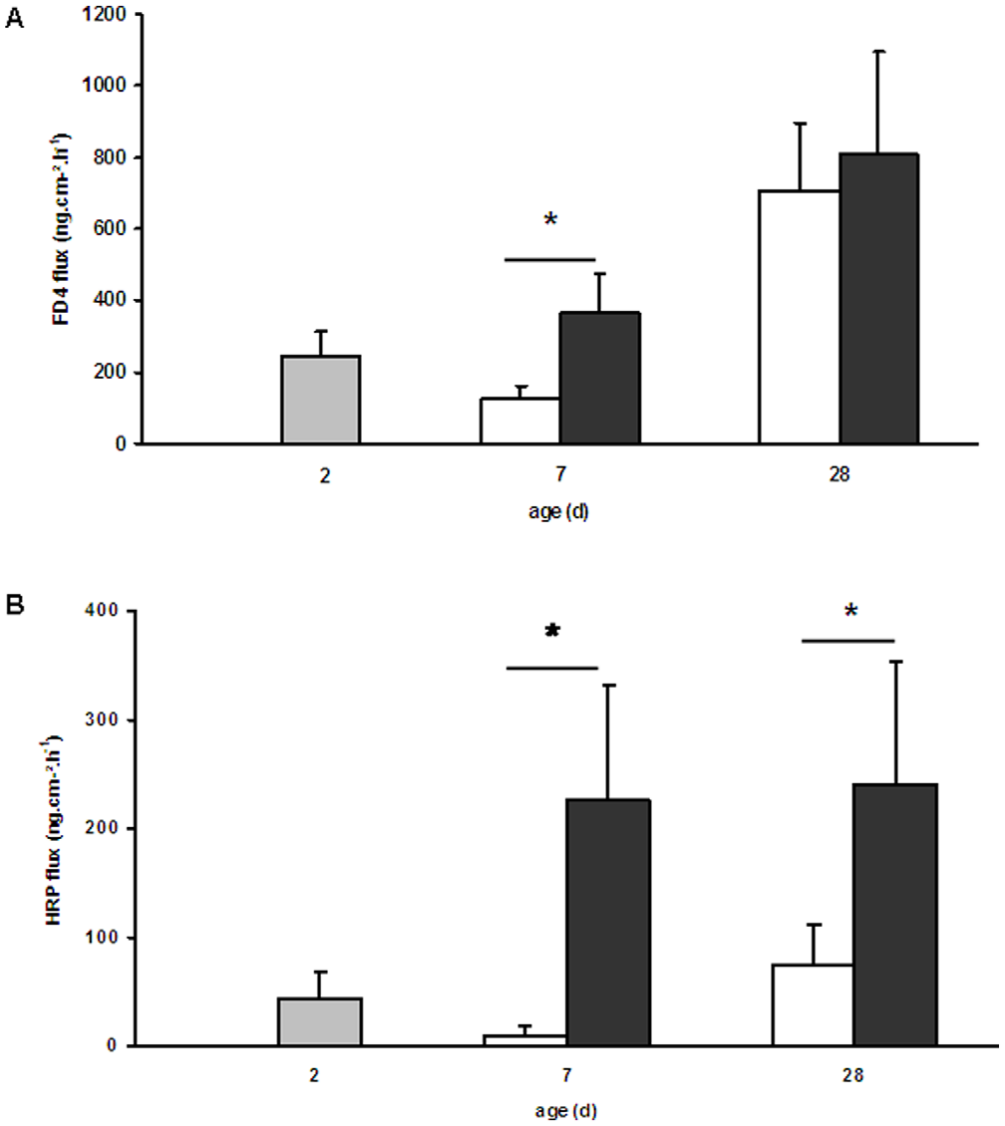
Ileal ex vivo permeability of NP and HP piglets. Permeability to FD-4 (A) and HRP (B) in the ileum of 2-d old piglets (grey bars) and of NP (open bars) and HP (solid bars) piglets at 7 and 28 days of age was determined in Ussing chambers. Values are means ± SE, n = 5–6. *, *P*<0.05. The HP formula induced an increase of FD-4 permeability at d 7 and HRP permeability at both d 7 and d 28 in the piglet ileum.

The effect of the HP formula on the physical barrier formed by epithelial cells was assessed by the measurement of *in vivo* bacterial translocation. Bacterial translocation to the lower mesenteric lymph nodes or to the spleen did not vary significantly between the two formulas, irrespective of age (data not shown). Since bacterial translocation reflects both epithelial barrier function and GALT capacity to neutralize bacteria, we also measured ileal permeability to small probes (FD-4) or macromolecules (HRP) *ex vivo* in Ussing chambers to evaluate specifically epithelial barrier function. The HP formula induced drastic changes in ileal permeability since FD-4 fluxes across mucosa was 2.9-fold higher in HP compared to NP piglets at d 7 (P = 0.03, Figure 1 A) and HRP fluxes 24-fold and 3-fold higher in HP than NP piglets at d 7 and 28 respectively (P = 0.03 and 0.05 respectively, Figure 1 B).

### Intestinal immune system during the neonatal period

The changes in ileal microbiota and epithelial barrier function induced by the HP formula prompted us to evaluate the development of the GALT. Since the neonatal period is an intensive period of immune cell recruitment in the lamina propria, we first assessed the effect of the HP formula on myeloid cells (CD172 positive cells) and T cells (CD3 positive cells) densities in the ileal lamina propria. No change was observed at d 7 (data not shown). At d 28 myeloid cells density was lower (73.9±10.4 vs 97.1±18.2 cells.mm^−2^, *P* = 0.04) and that of T cells was higher (285.6±89.0 vs 155.6±31.5 cells.mm^−2^, *P* = 0.007) in HP compared to NP piglets.

Together with immune cells expansion in the lamina propria, the neonatal period is an intense period of changes in expression of molecules involved in the recognition of bacteria by epithelial and immune cells, such as toll-like receptors (TLR) but also cytokines, either pro- or anti-inflammatory or regulatory. The HP formula reduced by 45% TLR2 mRNA level at d 28 of age (*P* = 0.002) but had no effect on TLR4 and TLR9 mRNA levels (Table 2). It also modified ileal cytokine profiles with reduction of the pro-inflammatory IL-1β mRNA level at d 7 (−60%, P = 0.03) but no effect on IL-6 and TNF-α mRNA levels (Table 2). Concerning regulatory cytokines, HP formula modified IL-10 mRNA level that tended to be higher in HP piglets at d 28 (+65%, *P* = 0.066) while TGF-β1 mRNA levels tended to be lower in HP compared to NP piglets at d 28 (−34%, *P* = 0.07, Table 2). Finally, IL-4 and IL-13 mRNA levels were too low to be detectable, irrespective of the group (Table 2).

**Table 2:** Toll-like receptors, cytokines and NF-κB subunits mRNA levels in the ileum of NP and HP piglets.

	d2	d7		d28	
		NP	HP	NP	HP
*Toll-like receptors*					
TLR 2	1.71±0.36	0.98±0.17	0.84±0.12	0.89±0.16	0.48±0.11*
TLR 4	0.72±0.11	0.70±0.12	0.71±0.09	0.70±0.03	0.66±0.12
TLR 9	0.43±0.08	0.83±0.15	1.19±0.25	1.24±0.26	1.09±0.27
*Cytokines*					
IL-1β	1.17±0.34	0.77±0.28	0.31±0.04*	0.33±0.05	0.36±0.17
IL-6	2.89±0.63	1.75±0.43	2.25±0.51	2.70±0.62	2.12±0.47
TNFα	0.77±0.21	0.77±0.13	0.80±0.12	2.10±0.98	1.45±0.52
IL-10	1.68±0.35	2.45±0.29	2.31±0.36	3.11±0.49	5.14±1.41#
TGF-β1	0.60±0.11	0.76±0.09	0.74±0.11	0.99±0.22	0.65±0.16#
IL-4	n.d.	n.d.	n.d.	n.d.	n.d.
IL-13	n.d.	n.d.	n.d.	n.d.	n.d.
*NF-kB sub-units*					
NF-kB1	0.23±0.03	0.36±0.06	0.22±0.03	0.30±0.04	0.38±0.09
NF-kB2	0.31±0.06	0.34±0.05	0.30±0.07	0.38±0.06	0.18±0.07*
RELA	0.94±0.10	1.10±0.15	1.31±0.28	1.11±0.14	0.28±0.07*
NF-kBIA	0.55±0.12	0.40±0.13	0.35±0.10	0.47±0.08	0.32±0.07*

Values are means ± SE, n = 6.

*P<0.05.,

# P≤0.07, n.d. not detectable.

Ileal NF-κB signaling pathway was also altered by the HP formula since we observed a down-regulation of NF-κB2 (coding the NF- κB p100 sub-unit), RELA (coding the NF- κB p65 sub-unit) and NFκBIA (coding IκBα) gene expressions at d 28 (−53%, P = 0.003; −75% P = 0.03 and −32%, P = 0.03 respectively, Table 2).

These different changes in the immune system were not associated with any difference in MPO activity in the ileal mucosa at any age (d 7: NP 1.64±0.93 and HP 2.60±0.57 U/mg mucosa; d28: NP 3.31±0.91 and HP 4.68±0.60 U/mg mucosa, P>0.05).

### Plasma IgG and haptoglobin concentrations during the neonatal period

**Figure 2:**
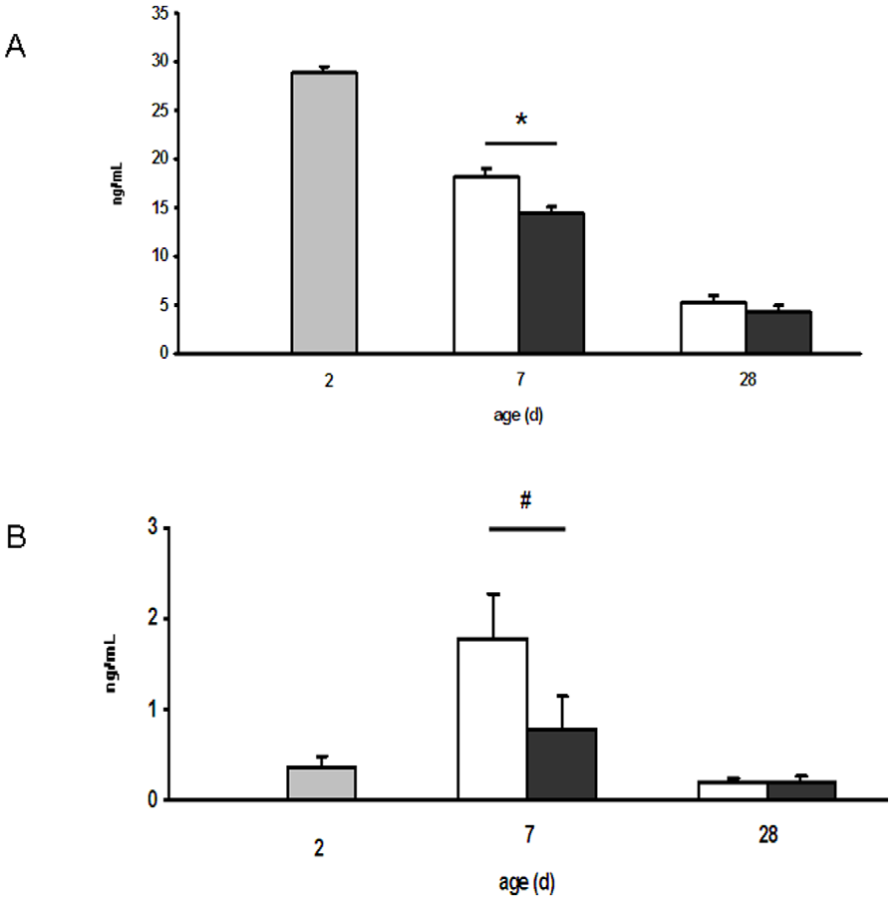
IgG and haptoglobin concentrations in the plasma of NP and HP piglets. IgG (A) and haptoglobin (B) concentrations were determined in the plasma of 2-d old piglets (grey bars) and of NP (open bars) and HP (solid bars) piglets at 7 and 28 days of age. Values are means ± SE; n = 5–6. *, *P*<0.05 ; #, P≤0.06. HP piglets exhibited lower plasma concentrations of IgG and haptoglobin than NP piglets at d 7.

We sought to see if the modifications of local ileal immune system development induced by the HP formula could have an impact at the systemic level. Plasma IgG and haptoglobin concentrations were lower (tendency for haptoglobin) in HP compared to NP piglets at d 7 (P = 0.03 and 0.06 respectively, Figure 2).

### Ileal sensitivity to inflammation later in life

The modifications in microbiota, epithelial barrier function and ileal immune system development observed during the neonatal period in this first experiment encouraged us to investigate if the HP formula had long-term consequences on intestinal barrier function and sensitivity to bacterial products in adult life in a follow-up experiment. Ileal permeability was not different between the two groups (FD-4: NP 451±96 vs HP 390±98 ng.cm^−2^.h^−1^ and HRP: NP 32±11 vs HP 26±11 ng.cm^−2^.h^−1^, P>0.05). TLR and cytokine mRNA levels were also not different between the two groups (Table 3).

**Table 3:** Toll-like receptors and cytokines mRNA expression at d160 of life.

	NP	HP
*Toll-like receptors*		
TLR2	1.56±0.16	1.79±0.34
TLR4	0.77±0.06	0.86±0.14
TLR9	1.11±0.17	1.56±0.43
*Cytokines*		
IL-1β	0.98±0.07	0.89±0.11
TNFα	0.95±0.18	0.86±0.17
IL-10	1.24±0.18	0.90±0.10
TGF-β1	1.00±0.10	1.10±0.18

**Figure 3:**
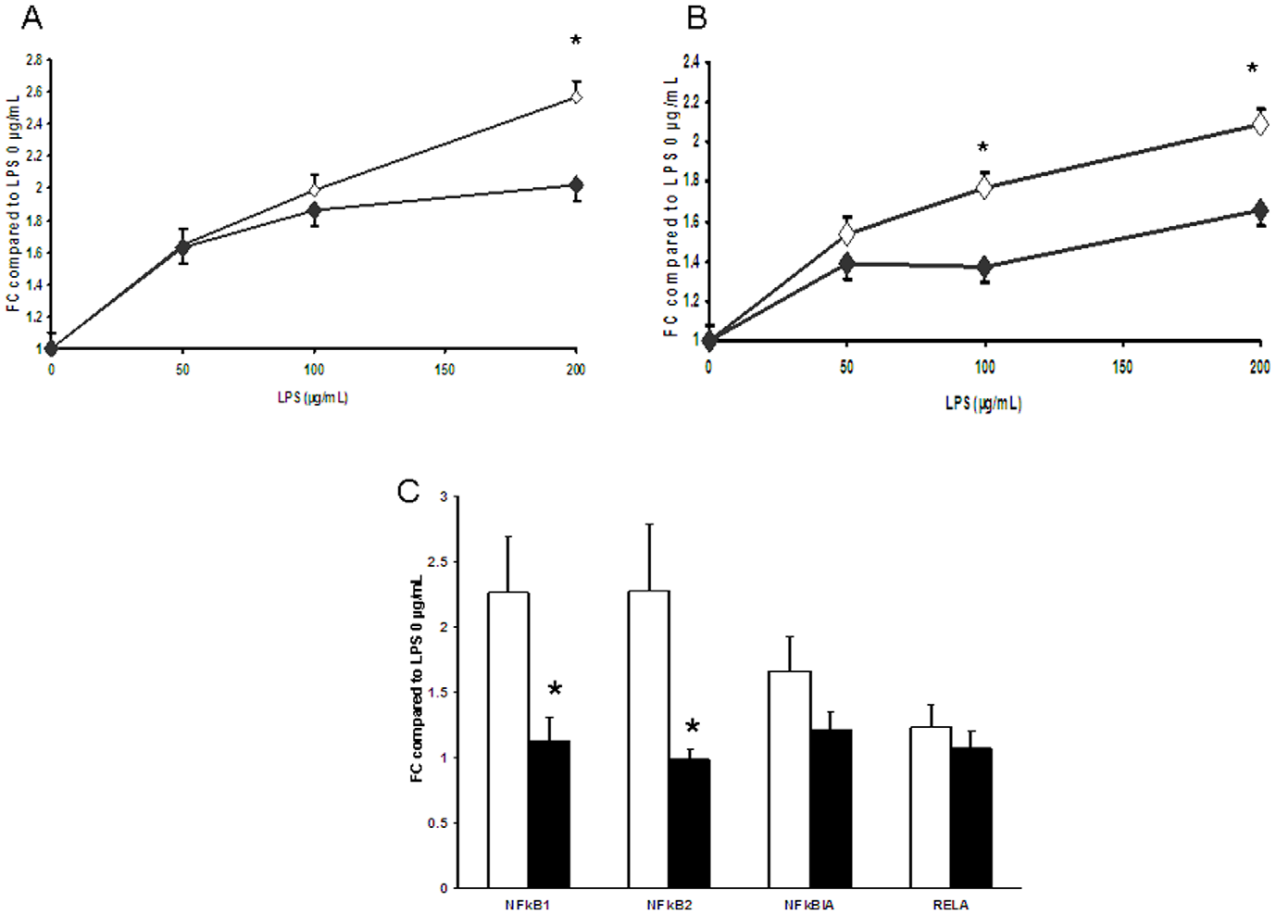
Ileal explant responses to LPS of NP and HP piglets at d 160 of life. TNFα (A) and IL-8 (B) release by ileal explants of 160 day-old piglets fed either the NP (open squares) or the HP (solid squares) formula from d 2 to d 28 of life was measured in response to different doses of LPS. NF-κB subunits mRNA levels were measured in explants stimulated with 200 µg/mL of LPS and compared to that of non-stimulated explants (C). Values are means ± SE; n = 8–10. *, *P*<0.05., FC fold-change. Ileal explants from HP pigs exhibited less cytokine release in response to high doses of LPS. Moreover, while NF-κB1 and 2 mRNA levels were increased after challenge with 200 µg/mL LPS in explants from NP pigs, this increase did not occur in explants from HP ones.

Ileal explants challenged with different doses of LPS were used to evaluate changes in the sensitivity of the mucosa towards bacterial products. Basal levels of IL-8 and TNFα in explants culture not stimulated with LPS were not different between the two groups (data not shown). Addition of LPS (50 to 200 µg/mL) significantly increased the level of cytokines released in the medium. However, the ileum of HP pigs exhibited lower levels of TNFα and IL-8 secretion in response to LPS (Figure 3 A–B). The challenge with LPS induced an increase of NF-kB1 and NF-kB2 mRNA levels in the ileal explants from NP pigs compared to the unchallenged explants. However, such an increase was not observed in explants from HP pigs (Figure 3 C). TLR4 mRNA levels after the LPS challenge were under the threshold of detection, irrespective of the group.

## Discussion

The major finding of our study is that feeding piglet neonates a high protein formula reduces the ileal inflammatory response to LPS in adulthood. This altered response followed early diet-induced changes in the developmental profile of intestinal microbiota, barrier function and immune system. Indeed feeding neonate piglets a HP formula increased bacterial concentrations in the ileum while decreasing the number of adherent *Lactobacilli* to the mucosa, enhanced ileal permeability to small and large molecules and seemed to accelerate gut immune system post-natal development.

High protein diets are known to increase satiety in several experimental adult animal model and also in humans [15]. Conversely, in the present study, the HP formula increased piglet growth. Energy intake, calculated on a metabolic weight basis, was not different between the two groups. Analysis of carcass composition of these piglets revealed an increase deposition of protein (+27% of protein) and reduction of adiposity (−36% of fat) in HP piglets [16]. Perirenal and subcutaneous adipose tissue characteristics and lipid metabolism were also modified by the HP formula [16]. The increase in piglet growth rate during the neonatal period in the present study agrees with the conclusion of a systematic review indicating that high protein intake in neonatal formula accelerates growth of low birth weight infants [17]. More recently, a large randomized trial concluded that infants receiving higher protein content in infant formula during the first year of life exhibited a greater growth [5]. However, other studies failed to show such effect [18], [19]. The discrepancy between the effect of a high protein diet in adults and in neonates might account for by the difference in protein needs at those two periods of life since protein requirement is the highest in the first month of life [20].

The high protein milk formula significantly increased bacterial counts in ileal digesta at d 7 in HP piglets, thus accelerating microbiota colonization during the neonatal period. Other studies in adult have shown a proliferating effect of dietary proteins or amino acids on intestinal microbiota [11], [12]. The higher load of dietary protein may lead to incomplete protein digestion in the small intestine (especially in immature piglets) and a significant part of alimentary and endogenous protein may reach the distal ileon and large intestine and be degraded by microbiota proteases and peptidases [21]. Despite this increase in bacterial concentrations in the luminal contents, the number of bacteria adherent to the mucosa did not change. On the contrary, the number of *Lactobacillus* adherent to the mucosa even decreased at d 7. That might therefore explain the absence of increase in bacterial translocation despite the huge increased permeability. Indeed adherence of bacteria to the mucosal surface rather than histological changes or alterations of epithelial permeability are the main factor determining bacterial translocation [22]. Moreover, lower adherence of *Lactobacillus* may explain the increase in ileal permeability that we have already observed in low birth weight piglets fed a high protein formula compared to adequate protein formula [23] since *Lactobacillus* are known to enhance gut barrier function [24], [25].

The mucosal immune system is almost completely absent at birth in the pig, and immune cells appear within the first two weeks of life to progressively increase with age [26]. The piglet must therefore rely on maternal immunoglobulins transferred to the neonate through the colostrum (mainly IgG) during the first 2 days of life [27]. The first antigen-presenting cells appear between 1 day and 2 weeks of life while T cells appearance is slower [27]. Collectively, our data are in favour of an acceleration of the immune system development with the HP formula. Indeed, the number of CD3-positive cells is increased in the ileal mucosa of HP piglets at d 28. Similarly, the age-related decrease of TLR2 and IL-1β mRNA levels and the age-related increase of IL-10 mRNA level seem to occur earlier in HP piglets. The role of dietary protein in the maturation of the immune system has been highlighted by a work on protein-deprived mice which had been fed from weaning till adulthood an amino-acid based diet. These adult mice had poorly developed GALT resembling suckling mice [14]. Moreover the commensal microbiota is a major stimulus for the development of the mucosal immune system as learnt from germ-free animals [28]. The excess load of antigens (dietary and bacterial) due to the increased permeability in the ileum seemed to have modulated the mucosal immune system development in our piglets. Antigen presenting cells may have captured and processed antigens to T cells resulting in a higher T cell density in HP comparatively to NP piglets [29].

We also observed reduced mRNA levels of actors of the NFκB pathway. NF-κB has a well-described pro-inflammatory activity. Indeed inflammatory bowel disease and experimental intestinal inflammation are characterized by NF-κB activation and increased expression of pro-inflammatory NF-κB target genes; accordingly, NF-κB inhibition protects against chronic intestinal inflammation and necrotizing enterocolitis in animal models [30]. However, recent findings suggest that NF-κB has not only pro-inflammatory but also tissue-protective functions [31]. Down-regulation of the various NF-κB sub-units at the mRNA level is commonly described as a regulatory mechanism to dampen [32] but also to augment long-term inflammatory responses [33] rending difficult to interpret the data. The ambiguity between a pro- or a anti-inflammatory profile is amplified by the fact that at the systemic level we observed a reduced plasma haptoglobin level in HP piglets while at the local level, ileal mucosa MPO activity was not influenced by the diet. Although additional experiments are required to further clarify the mechanism of intestinal adaptation to a high protein formula, the down-regulation of NF-κB sub-units mRNA levels may be driven both by amino acids, as described for glutamine [34], [35] and by specific commensal species [36].

Our data provide evidences for a long-term impact of neonatal nutrition in challenging conditions. The HP formula had no impact on ileal basal physiology later in life with normalized barrier function and cytokine profile. However, when challenged with high doses of LPS, the ileal mucosa of HP pigs secreted less pro-inflammatory cytokines than that of NP pigs. There was also no increase in NF-κB1 and 2 mRNA levels in response to LPS in explants from HP pigs as observed in NP ones. Bacterial cell surface LPS interacts with mucosal TLR4 to stimulate transcription and translation of pro-inflammatory cytokines (TNF, IL-6, IL-8) via the activation of the transcription factor NF-κB [37]. Ileal explants of HP pigs did respond to LPS stimulation since the level of pro-inflammatory cytokines released in the medium was significantly increased with LPS, irrespective of the LPS dose, compared to the un-stimulated condition. However, while explants from NP pigs exhibited a nice dose-response to LPS, cytokines release in HP explants was lower than that of NP pigs for the highest doses of LPS. TLR4 mRNA levels in the ileal mucosa were not different between the two groups, Toll-like receptor-4 mRNA but also the surface protein expression are down-regulated after LPS challenge in different immune cells [38], [39]. Accordingly, TLR4 mRNA was undetectable in our ileal explant 20 hrs after the LPS challenge, irrespective of the dietary group. This suggests that TLR4 was not involved in the differential response to LPS observed between the two dietary groups. Activation of NF-κB pathway after LPS challenge is an immediate response [32], [33]. A recent paper shows NF-κB pathway activation within 1 h followed by increase in NF-κB1 mRNA levels 8 h after the challenge in neuronal cells [40]. Down-regulation of the mRNA levels of NF-κB sub-units as observed after 20 h of LPS challenge in the HP group might be a mean to dampen inflammatory response and explain the lower concentration of cytokines measured in supernatants of explants form HP pigs.

Adult intestinal mucosa is tolerant to LPS, as demonstrated by the lower IL-8 secretion in response to LPS of adult intestinal tissue compared to foetal or neonatal ones [34], [41]. Although the exact mechanisms are not all unravelled, this ensures tolerance to the resident microbiota in adults. In the present study, ileal inflammatory response to LPS was similar at the doses of LPS usually used to demonstrate tolerance to LPS, suggesting that tolerance to LPS was not altered. The difference between the two groups was observed at high doses of LPS. Although it is difficult to extrapolate these *ex vivo* data to an *in vivo* situation, one can speculate that the reduced inflammatory response to high doses of LPS could have dramatic consequences in case of need of sustained response to inflammatory challenge *in vivo*. There is clearly a need for further investigations to understand how the impaired development of intestinal immune system early in life can have consequences later in life. A recent study has provided evidence of delayed effects of changing early microbial colonization of the gut on mucosal immune functions in weaned pigs [42]. In addition a neonatal LPS challenge was reported to amplify the immune response to experimental TNBS-induced colitis in rats [43]. Corroborating these findings we demonstrated that early modulation of ileal microbial colonization, epithelial barrier and immune development through nutrition influences ileal immune response to LPS challenge in adults.

In conclusion, our study demonstrates that the level of protein in formulas does have immediate consequences on ileal physiology. Furthermore, such high protein formulas also have delayed consequences on ileal immune response later in life. Whether this effect is directly linked to the higher protein content or also partly to the decrease in fat and carbohydrate content to keep formula energy constant and the mechanisms remain to be investigated.

## Materials and Methods

### Ethic statement

The experimental protocol was designed in compliance with recommendations of the French law (Décret: 2001-464 29/05/01) and EEC (86/609/CEE) for the care and use of laboratory animals under the certificate of authorization to experiment on living animals n°7676.

### Animals and experimental design

The experiments were performed on 54 crossbred (Piétrain×(Large White×Landrace)) piglets from the experimental herd of INRA (Saint-Gilles, France) in two separate experiments (short-term and long-term effects). We used low birth weight piglets (birth weight 0.90±0.02 kg) because of their relative intestinal immaturity [44] to maximize the chance to see an effect of dietary proteins. In the first experiment (short-term effects), piglets were separated from sows after colostrum intake at 2 days of life (d 2). A first group of 6 piglets from 6 litters was sacrificed at d 2 as initial controls. Then 12 pairs of littermates from 12 different litters were randomly allocated at d 2 to two artificially reared groups. One piglet of each pair received a normoprotein formula (NP) and the other one a high protein formula (HP) (see section below for feeding procedure details). The use of littermates in each pair ensured that the genetic but also microbiological backgrounds within the piglet pair would be close. Between d 2 and d 7, piglets were placed in incubators (33°C, 60 % humidity), and bottle-fed 9 times a day at 2 h intervals between 7 a.m. and 11 p.m., plus an extra meal at 3 a.m.. At d 7, 6 pairs of piglets were sacrificed, while 6 other pairs were transferred into individual cages equipped with an automatic device delivering milk 10 times a day (same schedule) in a temperature-controlled room at 30°C until d 28 (commonly the end of the suckling period in pig breeding). Piglets were sacrificed at d 28.

In the second experiment (long-term effects), 6 pairs of males and 6 pairs of females were selected at birth and followed exactly the same feeding protocol from d 2 to d 28 than in the first experiment. At d 28, piglets were weaned. They were kept in individuals cages until d 160 and fed the same diet for both groups (see post-weaning feeding procedure below).

For both experiments, clinical observations were recorded daily. Piglets were weighed daily from d 2 to d 6 then twice a week until d 160. Feed intake was recorded daily.

### Animal feeding

A NP formula supplying the same amount of nutrient as sow milk [45] was formulated using whey protein concentrate, skimmed milk powder and potassium caseinate as main sources of proteins. The HP formula was designed to provide a 40% higher amount of protein per day but the same casein/whey protein ratio and fat to carbohydrate ratio (Table 4). The increase in protein content of the HP formula was achieved by increasing the amount of whey protein concentrate and potassium caseinate. The individual daily milk quantity offered to the piglets was calculated to provide 1305 kJ.kg^−1^ of metabolic weight (body weight^0.75^) per day. The amount of protein offered to the HP piglets was progressively increased (30% higher than NP piglets from d 2 to d 7 and 40 % higher than NP piglets from d 8 onwards). This was achieved by offering the HP piglets a milk formula prepared by mixing NP and HP formulas (30/70 v/v) from d 2 to d 7. Both formula powders were manufactured by the Laiterie de Montaigu (Montaigu, France). Milk formulas were prepared daily from powders, maintained at 4°C, and then warmed up before meal.

**Table 4:** Composition of neonatal formula.

	NP	HP
*Ingredients (g/100 g powder)*		
Lactose	19.7	21.3
palm oil	14.7	13.0
milk fat	14.7	13.0
whey protein concentrate	14.4	20.3
skimmed milk powder	8.9	-
potassium caseinate	8.4	14.8
oleic sunflower oil	5.6	5.0
soybean oil	4.1	3.7
tricalcium phosphate	2.5	2.4
L-proline	1.7	1.5
L-glutamic acid	1	1
L-arginine	0.7	0.6
L-glycine	0.6	0.5
canola oil	0.6	0.6
disodium phosphate	0.5	0.3
vitamin and mineral premix^1^	0.5	0.5
L-valine	0.3	0.3
fish oil	0.3	0.3
arachidonic acid	0.3	0.3
L-histidine	0.2	0.2
magnesium chloride	0.2	0.2
choline bitartrate	0.2	0.2
*Composition (as fed)*		
Energy (kJ/kg BW^0.75^/d)	1305	1305
Protein (g/kg BW^0.75^/d)	14.1	20.0
Fat (g/kg BW^0.75^/d)	22.6	20.7
Carbohydrate (g/kg BW^0.75^/d)	13.5	12.2
Protein/energy (g/100 kcal)^2^	4.4	6.2

1 Providing per 100 g: vitamin A (retinol) 442.5 µg as retinyl acetate, vitamin D_3_ (cholecalciferol) 10.5 µg, vitamin E 0.77 mg as all-racemic α-tocopherol acetate, vitamin K_1_ (phylloquinone) 0.28 mg, vitamin C (sodium ascorbate ) 75 mg, vitamin B_1_ 0.56 mg as thiamine mononitrate, vitamin PP (nicotinamide) 6 mg, vitamin B_2_ (riboflavin) 1.10 mg, vitamin B_6_ (pyridoxine) 1.10 mg, folic acid 0.21 mg, calcium pantothenate 2.65 mg, vitamin B_12_ (cyanocobalamin) 2.30 µg, biotin 15.00 µg, iodine 100 µg as potassium iodide, Fe 11.9 mg as ferrous sulphate, Cu 2.00 mg as copper sulphate, Zn 11.60 mg as zinc sulphate, Mn 2.99 mg as manganese sulphate, Se 20 µg as sodium selenate.

2 The average protein/energy ratio in sow milk is 4.2 g/100 kcal.

In the second experiment, piglets were offered a standard post-weaning diet from d 28 to d 42 of life and then diets covering their specific protein and energy requirements but formulated to provide 30% of energy with lipids as observed in humans (Table 5). They were fed ad libitum.

**Table 5:** Composition of the weaning diet and the diets offered until d160 of life.

	weaning diet (d29–d42)	d43–d70 diet	d71–d160 diet
g/100 g dry matter			
Protein	19.5	21.6	19.0
Fat	6.6	11.8	11.8
Fiber	3.0	3.2	3.3
energy (MJ/kg)	10.61	11.15	11.16

### Tissue collection

Piglets were euthanized at d 2, d 7, d 28 and d 160 by electronarcosis and bleeding. At d 2, d 7 and d 28, blood was collected in heparinized tubes, centrifuged (4000 g, 10 min, 4°C) and plasma was stored at −20°C. Dissections were performed under aseptic conditions to sample the spleen and mesenteric lymph nodes from the lower intestine to evaluate bacterial translocation. Digestive contents and mucosa from distal ileum (15 cm proximal to the ileo-caecal valvula) were sampled under aseptic conditions for bacteria count. Adjacent segments of ileum were rinsed with cold saline and either placed into Ringer buffer (composition in mmol.L^−1^: Na^+^ 145, Cl^−^ 128, PO_4_^3−^ 0.32, Ca^2+^ 2, Mg^2+^ 1, HCO_3_^−^ 25, SO_4_^2−^ 1, K^+^ 6.3) at 4°C for immediate Ussing chamber analysis, or into RNA later (Applied Biosystems, Courbaboeuf, France) (cross-section of tissue) at 4°C for 24 h and then maintained at −20°C for later RT-PCR analysis. Five-centimeter adjacent segments were also rinsed and fixed in 4 % formaldehyde buffer for 24 h, dehydrated in ethanol, stored at 4°C before being embedded in paraffin. At d 160, segments of ileal mucosa were rinsed with cold sterile saline then either placed into Ringer buffer for Ussing chamber analysis or into RNA later (cross-section of tissue) at 4°C for 24 h and then maintained at −20°C for later RT-PCR analysis. An adjacent segment was rinsed twice with PBS supplemented with 1% fetal calf serum (FCS, Gibco, Invitrogen, Cergy-Pontoise, France) and 0.5 mM DTT (Sigma-Aldrich, St Quentin Fallavier, France), once with PBS then placed in a PBS/DMEM (3/1 v/v) solution containing 1% FCS, 5 µg.mL^−1^ gentamicin (Sigma-Aldrich) and 1.25 µg.mL^−1^ amphotericin B (Sigma-Aldrich) for immediate explants culture.

### Microbiological methods

Within 1 h after collection, mesenteric lymph node and spleen samples were weighed (50 to 500 mg) and homogenized (Kinematica, Polytron, Littau-Lucerne, Switzerland) in 1 mL of sterile 0.9 % NaCl. Ileal mucosa was weighed (10 to 500 mg) and gently rinsed with 10 mL sterile 0.9 % NaCl. It was then homogenized in 1 mL of sterile 0.9 % NaCl. Digestive contents were weighed (10 to 400 mg) and placed into 1 mL of sterile 0.9 % NaCl. Samples were then diluted at 1/1000. Ten microliters of each samples were plated on various selective and nonselective media to allow numeration of specific bacterial types. Chromogenic UTI Medium (Oxoid, Cambridge, UK) was used for the presumptive identification and differentiation of the main aerobes (*Enterobacteria*, *Lactobacillus*, *Enterococcus*, *Staphylococcus*). Columbia CAN agar +5 % sheep blood (BD Biosciences, Le Pont de Claix, France) was used for *Gram*+ bacteria, Schaedler agar +5 % sheep blood medium (BD Biosciences) for total aerobes and anaerobes, Schaedler agar with kanamycin/vancomycin (BD Biosciences) for selective culture of *Gram*- anaerobes. Man-Rogosa and Sharpe (MRS) medium (Difco, Detroit, USA) was used for *Lactobacillus*. The anaerobic atmosphere was obtained using anaerocult A (Merk, Darmstadt, Germany). Aerobic and anaerobic cultures were examined after 24 and 48 h of incubation, respectively. Bacterial counts of cultured mucosa and contents are expressed as the log colony-forming units (CFU) per gram of content or tissue. The count threshold was 10^2^ CFU.g^−1^. Lymphoid tissue translocation was expressed as percentage of positive tissues in each group.

### Ussing chamber

Intestinal tissues were stripped of longitudinal muscle and opened along the anti-mesenteric border then mounted in Ussing chamber (World Precision Instrument, Stevenage, UK). The chamber opening exposed 0.67 cm^2^ of tissue surface area to 8 mL of circulating oxygenated Ringer at 39°C. The serosal buffer contained 16 mmol.L^−1^ glucose osmotically balanced by 16 mmol.L^−1^ mannitol in the mucosal buffer.

The paracellular and transcellular passages were determined with FITC-4000 (FD-4, Sigma-Aldrich) and horseradish peroxydase (HRP Type II, Sigma-Aldrich), respectively. FD-4 and HRP (375 mg.L^−1^ each) were added to the mucosal buffer at t0. Five hundred microliters samples were collected at 30 min intervals for 120 min from the serosal buffer and replaced with Ringer-glucose buffer to maintain a constant volume within the chambers. Concentration of FD-4 in the samples was measured by fluorimetry (fluorimeter LB940 Mithras, Berthold Technologies, Thoiry, France) while concentration of HRP was determined using spectrophotometry (Multiskan spectrum, Thermo Labsystem, Midland, Canada) after enzymatic reaction using o-dianisidine as substrate (Sigma-Aldrich). Mucosal-to-serosal fluxes were then calculated and expressed as ng.cm^−2^.h^−1^.

### Myeloperoxidase (MPO) assay

Myeloperoxidase (MPO) activity was determined in ileal mucosa using an O-dianisidine-hydrogen hydroperoxide method [46] after MPO had been extracted from tissues according to Barreau et al. [47].

### Explant culture

Explant culture protocol was adapted from Coeffier et al. and Nanthakumar et al. [41], [48]. Briefly, ileal mucosa was cut in small pieces of 10–15 mg which were let in a DMEM solution containing 1% FCS, 5 µg.mL^−1^ gentamicin, 1.25 µg.mL^−1^ amphotericin, 100 U.mL^−1^ penicillin (Gibco), 100 µg.mL^−1^ streptomycin (Gibco), 2 mM L-glutamine (Gibco) and 1 mM sodium pyruvate (Gibco) for 2 h under a 95% CO_2_-5% O_2_ atmosphere at 37°C. Explants were then transferred into the same DMEM solution as previously described but without FCS (2 pieces i.e. 20–35 mg of mucosa per mL) and either 0, 50, 100 or 200 µg/mL LPS (Sigma-Aldrich) and incubated for 20 h under a 95% CO_2_-5% O_2_ atmosphere at 37°C. At that time, supernatants were immediately frozen and stored at −20°C for later cytokines analysis and tissues were frozen at −80°C in Trizol reagent (Invitrogen) for later mRNA extraction. IL-8 and TNFα concentrations in the supernatants were determined by ELISA (R&D Systems Europe, Lille, France).

### Quantitative RT-PCR analysis

Total RNA was extracted from tissues with Trizol reagent and chloroform, precipitated by isopropanol. Ethanol was then used to wash the precipitate, which was hydrated with 100 µL of water. Extracted RNA were quantified by spectrophotometry (Nanodrop, Wilmington, USA) and treated by the DNA-free kit (Ambion, Austin, USA). Their quality was assessed by fluorimetry by the RNA 6000 nano LabChip® kit in 2100 Bioanalyser (Agilent Biotechnologies, Santa Clara, USA). Reverse transcription was performed with 2 µg of total RNA using the High capacity cDNA archive kit (Applied Biosystems). Obtained cDNA were amplified using specific primers for TLR2, TLR4, TLR9, IL-1β, IL-6, TNF-α, IL-10, TGF-β, NF-kB1, Nf-kB2, RELA, NF-kBIA, IL-4 and IL-13 genes (Table S1). The real time quantitative PCR were performed with the obtained cDNA, both forward and reverse primers (500 nmol.L^−1^), Uracyl-DNA-glycosylase (Invitrogen) and SYBR® Green I PCR core reagents (Applied Biosystems) in an ABI PRISM 7000 Sequence Detection System instrument (Applied Biosystems). Forty cycles of amplification were performed (denaturation at 95°C for 15 s, annealing and extention at 60°C for 1 min). Amplification product specificity was checked by dissociation curve analyses. To determine the efficiency of each primer, a standard curve was established from serial dilutions of a RT product sample pool. Then, for each sample, the relative amount of the target RNA was determined by comparison with the corresponding standard curve. Finally, the transcript level was normalized to the transcript level of GAPDH (housekeeping gene, see Table S1 for primer sequence) in the same sample.

### Fluorescence immunohistochemistry

The postnatal development of gut immune cells was focused on CD172^+^ myeloid cells, i.e. granulocytes, monocytes, macrophages and dendritic cells and CD3^+^ T cells. Five-micrometer paraffin sections were rehydrated in toluene (3 washes of 10 min) and in ethanol (3 washes of 5 min). To unmask leukocyte antigens, tissue sections were incubated twice in 10 mM Tris-EDTA buffer, pH 9.0, for 3–5 min at 650 W using a microwave oven (VIP20, Whirlpool Philips, Sweden). Sections were left 20 min at room temperature after each microwave treatment. After one wash for 5 min in PBS, sections were simultaneously incubated with FITC-conjugated anti-porcine CD3 (clone PPT3, IgG1 isotype, Beckman Coulter, Krefeld, Germany) at 20 mg.L^−1^ and red phyco-erythrin anti-porcine CD172 (clone 74-22-15, IgG1 isotype, Beckman Coulter) at 10 mg.L^−1^. In parallel, conjugated mouse F(ab′)_2_ IgG1 isotype controls (Southern Biotechnology Associates, Birmingham, USA) were used as negative controls. One ileal section of each animal was observed using a Nikon ECLIPSE E400 epifluorescence microscope (Nikon, Tokyo, Japan). Ten representative areas of 0.14 mm^2^ each were recorded with CCD camera (Nikon DXM 1200, 5.3 version,Nikon) and CD3^+^ cells and CD172^+^ cells were counted by NIS software (Nikon Instruments Inc., Melville, USA). Results are expressed as positive cell number.mm^−2^.

### Plasma IgG and haptoglobin concentrations

IgG levels were determined in the plasma by quantitative ELISA sandwich using a pig dedicated ELISA kit (Bethyl Pig IgG ELISA quantitation kit, Interchim, Montluçon, France). Haptoglobin concentrations were determined in the plasma by a pig dedicated colorimetric kit (Tridelta Ltd, Maynooth, Ireland).

### Statistical analyses

Statistical analyses were performed using the General Linear Model procedure of Statistical Analysis Systems software (SAS Institute, Cary, NC, USA), testing the piglet pair, age, diet and age×diet interaction, with t-test as a subsequent multiple comparisons when appropriate. Translocation data were analysed using the non parametric chi2-test. All results are presented as means ± SE. Differences between groups were declared significant at *P*<0.05.

## Supporting Information

Table S1 **Primers used in real-time PCR.** S = sense primer, AS = anti-sense primer. The primers were designed using Primer Express Software (Applied Biosystems) based on *sus scrofa* published nucleotide sequences (Iccare, http://bioinfo.genopole-toulouse.prd.fr/Iccare/)
(DOC)
