# Supplementary material for: The Level of Protein in Milk Formula Modifies Ileal Sensitivity to LPS Later in Life in a Piglet Model
Source: PLoS One. 2011 May 9;6(5):e19594. doi: 10.1371/journal.pone.0019594 (PMC3090415; doi:10.1371/journal.pone.0019594)
Supplement: Table S1 — Primers used in real-time PCR. S = sense primer, AS = anti-sense primer. The primers were designed using Primer Express Software (Applied Biosystems) based on sus scrofa published nucleotide sequences (Iccare, http://bioinfo.genopole-toulouse.prd.fr/Iccare/) (DOC) [file pone.0019594.s001.doc]

| Gene |  | Primer sequence | Accession number |
| --- | --- | --- | --- |
| TLR-2 | (S)  (AS) | 5’-GACTGGCCGGAGAACTACCT-3’  5’-TGAGCAGGAGCAGCAGGAA-3’ | AB208696 |
| TLR-4 | (S)  (AS) | 5’-CAAGGACCAGAAGCAGCTCC -3’  5’-GACGGCCTCGCTTATCTGAC-3’ | [AB188301](http://www.ncbi.nlm.nih.gov/entrez/viewer.fcgi?db=nucleotide&val=73760381) |
| TLR-9 | (S)  (AS) | 5’-GGACCTCAGCTACAACAGCC-3’  5’-CGGCTATGGATGTCATTGTG-3’ | [NM213958](http://www.ncbi.nlm.nih.gov/entrez/viewer.fcgi?db=nucleotide&val=47522745) |
| IL-1β | (S)  (AS) | 5’-GTGATGGCTAACTACGGTGACAA-3’  5’-CTCCCATTTCTCAGAGAACCAAG-3’ | M86725 |
| TNF-α | (S)  (AS) | 5’-ATCGGCCCCCAGAAGGAAGAG-3’  5’-GATGGCAGAGAGGAGGTTGAC-3’ | M29079 |
| IL-6 | (S)  (AS) | 5’-GTCGAGGCTGTGCAGATTAGT-3’  5’-TTCTGTGACTGCAGCTTATCC-3’ | M80258 |
| IL-10 | (S)  (AS) | 5’-CGGCGCTGTCATCAATTTCTG-3’  5’-CCCCTCTCTTGGAGCTTGCTA-3’ | L20001 |
| TGF-β1 | (S)  (AS) | 5’-TACGCCAAGGAGGTCACCC-3’  5’-CAGCTCTGCCCGAGAGAGC-3’ | NM214015 |
| IL-4 | (S)  (AS) | 5’-CGGTGACGACGTCTTTGCT-3’  5’-CCCGGCAGAAGGTTTGCT-3’ | X68330 |
| IL-13 | (S)  (AS) | 5’-GTCATTGCTCTCACCTGCTT-3’  5’-TTGGTGTCTCGGATGTGCTT-3’ | AF385626 |
| NF-κB1 | (S)  (AS) | 5’-TCGCTGCCAAAGAAGGACAT’3’  3’-AGCGTTCAGACCTTCACCGT-5’ | DQ834921 |
| NF-κB2 | (S)  (AS) | 5’-ACCAGCGTCATTGAGCAGATAG-3’  3’-CCCGTGATGACTGCCAAGT-5’ | AY163838 |
| NF-κBIA | (S)  (AS) | 5’-CCACACTTCAACAAGAGCGA-3’  3’-TCTTTGGGTGCTGATGTCAA-5’ | BC166886 |
| RELA | (S)  (AS) | 5’-CGAGAGGAGCACGGATACCA-3’  3’-GCCCCGTGTAGCCATTGA-5’ | AK303262 |
| GAPDH | (S)  (AS) | 5’-CATCCATGACAACTTCGGCA-3’  5’-GCATGGACTGTGGTCATGAGTC-3’ | AF017079 |
